# 24-h movement behaviours in Spanish youth before and after 1-year into the covid-19 pandemic and its relationship to academic performance

**DOI:** 10.1038/s41598-022-21096-5

**Published:** 2022-10-05

**Authors:** Miguel Angel Tapia-Serrano, David Sánchez-Oliva, Javier Sevil-Serrano, Adilson Marques, Pedro Antonio Sánchez-Miguel

**Affiliations:** 1 Departamento de Didáctica de la Expresión Musical, Plástica y Corporal, Grupo Análisis Comportamental de la Actividad Física y el Deporte (ACAFYDE), Facultad de Formación del Profesorado, Universidad de Extremadura, Av. de la Universidad, s/n, 10004 Cáceres, Spain; 2 Departamento de Didáctica de la Expresión Musical, Plástica y Corporal, Grupo Análisis Comportamental de la Actividad Física y el Deporte (ACAFYDE), Facultad de Ciencias del Deporte, Universidad de Extremadura, Av. de la Universidad, s/n, 10004 Cáceres, Spain; 3 CIPER, Faculdade de Motricidade Humana, Universidade de Lisboa, 1499-002 Lisbon, Portugal

**Keywords:** Systems biology, Diseases, Health care

## Abstract

Most studies have shown a decline in the adherence to 24-Hour Movement Guidelines because of Covid-19 lockdown. However, there is little evidence regarding changes 1-year after the pandemic in these guidelines and their possible impact on academic performance. The study aims were: (1) to examine the possible changes in 24-Hour Movement Guidelines for youth (i.e., at least 60 min per day of moderate-to-vigorous physical activity, ≤ 2 h per day of recreational screen time, and 9 to 11 h of sleep per day for children and 8 to 10 h for adolescents) before and after 1-year into the Covid-19 pandemic, and (2) to examine the possible changes in the relationship between 24-Hour Movement Behaviours (physical activity, screen time, and sleep duration) and academic performance before and after 1-year into the Covid-19. This is a repeated cross-sectional study in two different samples of young Spanish at different times. Firstly, a total of 844 students (13.12 ± 0.86; 42.7% girls) completed a series of valid and reliable questionnaires about physical activity levels, recreational screen time, sleep duration and academic performance before Covid-19 pandemic (March to June 2018). Secondly, a different sample of 501 students (14.39 ± 1.16; 55.3% girls) completed the same questionnaires 1-year after Covid-19 pandemic (February to March 2021). Adherence to the three 24-Hour Movement Guidelines was significantly lower 1-year after into the Covid-19 pandemic (0.2%) than before the pandemic (3.3%), while adherence to none of these three recommendations was significantly higher 1-year after the Covid-19 pandemic (66.3%) than before the pandemic (28.9%). The positive relationship between physical activity levels and academic performance was no longer significant after 1-year into Covid-19 pandemic (β = − 0.26; *p* < 0.001). 1-year after Covid-19 pandemic, the relationship between recreational screen time (β = − 0.05; *p* > 0.05) and sleep duration (β = 0.05; *p* < 0.001) with academic performance did not change compared to pre-pandemic. The results suggest that 24-Hour Movement Behaviours have worsened among young people 1-year after Covid-19 pandemic compared to pre-pandemic period. Moreover, the physical activity benefits associated in terms of academic performance seem to have disappeared because of the Covid-19 pandemic. Therefore, there is a public health problem that requires priority and coordinated action by schools, policy makers, and researchers to mitigate the adverse effects of the pandemic on 24-Hour Movement Behaviours.

## Introduction

On 11th March 2020, the World Health Organization^1^ declared the outbreak of coronavirus disease 2019 (Covid-19) as a global pandemic. In early March, Spain experienced an initial increase of 4,200 Covid-19 cases, reaching a peak of 9,181 cases on 26th March 2021^2^. To stop its spread, on 14th March 2020, the Spanish government (and other nations worldwide) declared a state of alarm followed by lockdown measures (from 14th March to 4th May 2020), limiting people's mobility (except for work and essential activities such as going to the supermarket or doctor), and forcing the closure of many institutions such as schools, high schools, and kindergartens. This also resulted in the cessation of organised sports and recreational activities and limited access to outdoor places such as playgrounds and parks^3,4^.

Restrictions or closure measures of Covid-19 meant prohibiting children and adolescents from leaving home for 6 or more weeks at a time, as well as replacing face-to-face lessons with home schooling and online learning activities^5^. A decrease in Covid-19 cases in Spain in the late spring and summer months led to a reduction in containment measures. In September 2020, most schools and high schools in Spain reopened their doors, using different safety measures such as 1.5 m distance, not sharing materials or the use of face masks. Sports and recreational activities were also resumed with new safety protocols^6^. Therefore, while all these public health restrictions were necessary to reduce the spread of Covid-19, the health measures adopted by Spain and other countries globally has been negatively related to physical, psychosocial, and cognitive health^7,8^.

Several authors have suggested that promoting a healthy lifestyle could prevent the adverse health effects of Covid-19^8^. Particularly, there is clear evidence that high physical activity levels^9^, low recreational screen time^10^, and optimal sleep duration^11^, contribute to the overall health and youth development. Meeting these three 24-Hour Movement Guidelines for children and adolescents (at least 60 min per day of moderate-to-vigorous physical activity, ≤ 2 h per day of recreational screen time, and 9 to 11 h of sleep per day for children and 8 to 10 h for adolescents) could even maximize those health benefits in young people^12^. However, the systematic review and meta-analysis conducted by Tapia-Serrano et al.^13^ reported that only 2.68% of adolescents met all three 24-Hour Movement Guidelines, while 28.59% did not meet any recommendation. The limited number of studies carried out in Spain indicated that between 1.7% and 5.4% of adolescents met all three recommendations, while between 8.7% and 10.2% did not comply with any recommendation^14,15^.

The scoping review conducted by Paterson et al.^16^ showed a decline of physical activity levels, an increase in recreational screen time, and irregular sleep patterns, particularly among adolescents, because of Covid-19-related restrictions. Particularly, in Spain, there were only three studies that compared the 24-Hour Movement Guidelines in children and adolescents before and during Covid-19 closure^17–19^. The results of these investigations showed negative effects of Covid-19 closure on meeting physical activity recommendations (34.6% to 60.0% before closure vs. 26.5% vs. 51.0% during closure) and sedentary behaviours, especially screen recreational behaviours guidelines (2.5% to 5.0% before closure vs. 1.8% vs. 2.4% during closure), but not on sleep duration guidelines (57.3% to 84.4% before closure 66.6% vs. 84.8% during closure). Nevertheless, most of the studies included in this scoping review examined changes in 24-Hour Movement Guidelines before and during Covid-19 lockdown^16^.

To our knowledge, there is only one study that have examined trends of 24-Hour Movement Guidelines before and after approximately 1-year into the Covid-19 pandemic^20^. In this study, there were significantly fewer adolescents meeting the three 24-Hour Movement Guidelines during the autumn 2020 than before Covid-19 (5.5% vs. 1.1%). In addition, a significantly higher number of adolescents did not comply with any of the three recommendations during the autumn 2020 than before Covid-19 (50% vs. 17.1%). Specifically, a decrease in physical activity and sleep duration recommendations was identified, while there was no change in screen time guidelines^20^. However, these findings are not extensible to the rest of the world, as they only looked at children and adolescents in Montreal (Canada). Therefore, further research is required to examine the long-term impact of Covid-19 on 24-Hour Movement Guidelines of youth^16^.

Moreover, a previous systematic review showed a negative effect of school closures on academic performance^21^. Given the adoption of a healthy lifestyle has been positively associated with brain development processes, cognitive function, and academic performance^22,23^, one would expect that the decline in 24-Hour Movement Guidelines would have negatively affected academic performance. Particularly, previous researches have shown that higher physical levels^9,24^, lower recreational screen time^25^, and optimal sleep duration^11^ have been positively and independently related to academic performance. To the best of our knowledge, no previous studies have examined the possible changes in the relationship between these 24-Hour Movement Behaviours (i.e., physical activity, recreational screen time, and sleep duration) and academic performance before and 1-year after into the Covid-19 pandemic and, therefore, further researches are also required. It is important to know whether this relationship may have been altered by the Covid-19 pandemic, as, for example, the type of physical activity may be different (e.g., safe distance, use of face masks, avoidance of sharing materials, etc.) or the quality of sleep may have been impaired. Therefore, perhaps the relationship between 24-Hour Movement Behaviours and academic performance could have been altered because of Covid-19 pandemic.

Thus, the first aim of this repeated cross-sectional study was to compare 24-Hour Movement Guidelines, separately and together, before (T1; March to June 2018) and 1-year after the Covid-19 pandemic (T2; February to March 2021) in two different subsamples of adolescents. Consistent with previous studies^17–19,26^ that have shown a decline in adherence with the 24-Hour Movement Guidelines before and during Covid-19 closure, is also expected to be lower 1-year after the Covid-19 pandemic. The second objective was to examine whether the relationship between 24-Hour Movement Behaviours and academic performance was different before and 1-year after the Covid-19 pandemic. Because a decline in adherence to the 24-Hour Movement Guidelines has been observed^21^, the first hypothesis of the study was that adherence to these recommendations has declined 1-year after the Covid-19 pandemic. With regard to the second hypothesis, because Covid-19 may have altered 24-Hour Movement Guidelines^16^, it is expected that the relationship with academic performance may have changed after 1-year into the Covid-19.

## Method

### Design and participants

**Figure 1:**
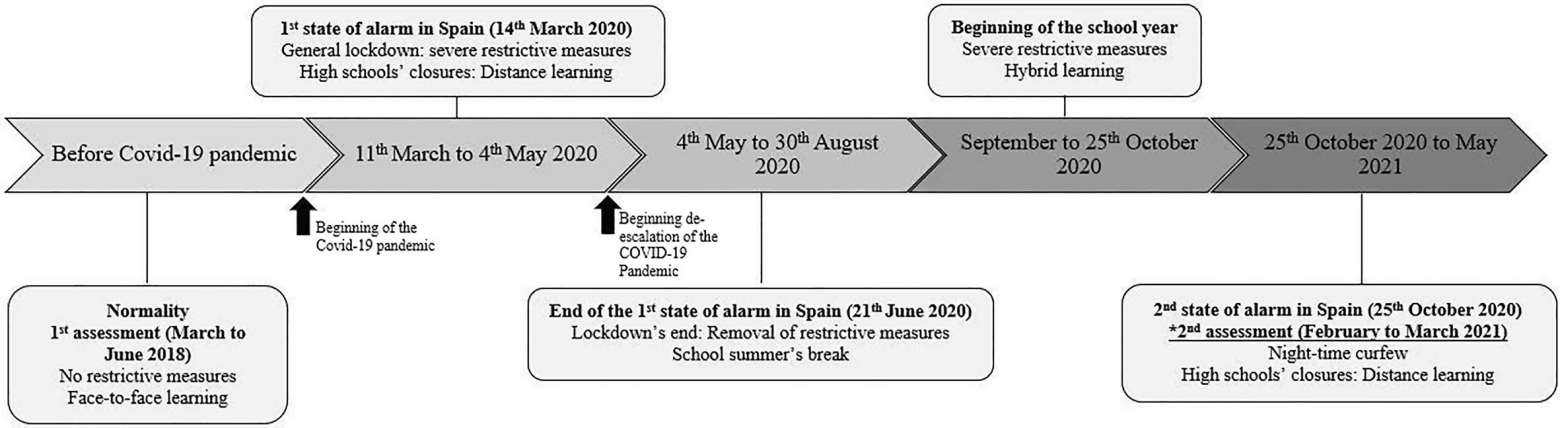
Turning of measurements before the covid-19 pandemic and for the years 2020 and 2021 for covid-19 restrictive measures in Spain.

A repeated cross-sectional study was carried out in two different samples of Spanish youths at different times (See Fig. 1). A simple random sampling was used to select the two sub-samples. Firstly, a total of 844 students (13.12 ± 0.86; 42.7% girls), aged 11 to 16 years, completed a series of valid and reliable questionnaires about physical activity levels, recreational screen time, sleep duration, and academic performance before the pandemic (March to June 2018; T1). Secondly, a different sample of 501 students (14.39 ± 1.16; 55.3% girls), aged 12 to 16 years, competed the same questionnaires 1-year after the Covid-19 pandemic (February to March 2021; T2), during the third state of alert in Spain^27,28^. During this period (the third state of alert started from 9th November 2020, until 9th May 2021 in Spain), several measures were put in place to prevent the spread of the virus: the movement of people in public spaces was limited between 11:00 PM and 6:00 AM; the capacity of public and/or sporting venues (e.g., gyms, sports centres) was limited, playgrounds in the parks were closed, and several federated sports activities were suspended (See Fig. 1 for more detail)^27,28^.

All data were collected in Extremadura, a region located in southwestern Spain. Both groups were similar in terms of age, sex, and socio-economic status, as all schools included belonged to neighbourhoods with similar socio-demographic characteristics^29^. This study was carried out in accordance with the Declaration of Helsinki and was approved by the Ethics Committee of the University of Extremadura (89/2016).

### Measures

Socio-demographic data, physical activity, recreational screen time, sleep duration, and academic performance were measured during the two assessments in the two sub-samples.

*Sociodemographic characteristics*. Student’s self-reported age (in years), sex (male/female), weight and height. The body mass index (BMI) of each participant was calculated using Cole’s international cut-off points based on their sex and age^30^.

*Physical activity*. Physical activity was assessed using the Spanish version of a self-reported questionnaire called Physical Activity Questionnaire for Adolescents (PAQ-A) validated by Martínez-Gómez et al.^31^. This questionnaire has shown to be a valid (the PAQ-A correlation with total physical activity [r = 0.39] and moderate and vigorous physical activity [r = 0.34] assessed by accelerometer) and reliable (α = 0.79 and Intraclass Correlation Coefficient [ICC] = 0.71) instrument to assess physical activity levels in Spanish youth aged 12–17 years. In the present study, Cronbach’s alpha of this scale was 0.87. The scale consists of nine items assessing participation in physical activity during the last seven days. Adolescents should respond to the frequency of physical activity participation in a list of activities such as physical education classes, school recess, at lunchtime, right after school, in the evening, and during the last weekend. Each response is scored from 1 to 5 using a Likert-type scale. The average of these scores results in the physical activity index score.

*Recreational screen time*. Recreational screen time was assessed using the Youth Leisure-Time Sedentary Behaviour Questionnaire (YLSBQ) validated by Cabanas-Sánchez et al.^32^. The questionnaire is a valid (r = 0.36) and reliable (ICC = 0.75) measure to assess sedentary recreational screen time among Spanish young people aged 8 to 18 years^32^. Students’ self-reported time spent on television, video games, computers, and mobile phones for both weekdays and weekend days. An average of recreational screen time was calculated for each screen-based behaviour in a ratio of 5:2 (e.g., [Daily TV viewing on weekdays × 5] + [Daily TV viewing on weekend days × 2]/7). The average daily recreational screen time was calculated by summing the different daily screen-based behaviours.

*Sleep duration*. Sleep duration was measured using a Spanish version of Pittsburgh Sleep Quality Index validated in 2003 by Macías and Royuela^33^. Students self-reported the time they usually go to bed and wake up on both weekdays and weekends. These questions have been shown as a valid (r = 0.45–0.90) and reliable (ICC = 0.71–0.99) measure to assess sleep duration in youths^34^. The average sleep duration was calculated using the following formula: ([Sleep duration on weekdays × 5] + [Sleep duration on the weekend days × 2]/7).

*Adherence to the 24-Hour Movement Guidelines*. Adherence to the 24-Hour Movement Guidelines established by Tremblay et al.^35^ was also calculated in this study. Children and youth aged 5–17 years should accumulate at least 60 min of moderate-to-vigorous physical activity per day (in the present study, we used the cut-off points of 2.75 in the PAQ-A^36^), spend less than two hours of recreational screen time per day, and sleep between 9 and 11 h per day (children aged 5–13) and 8 to 10 h per day (adolescents aged 14–17) to meet each of the three recommendations^35^.

*Academic performance*. Academic performance was assessed through school records at the end of the academic year. Academic performance was based on four subjects: first language (Spanish), second language (English), mathematics, and physical education. The grade point average (GPA) was then calculated as an average of the scores in these four subjects. Previous studies have used these subjects as indicators to assess academic performance^37,38^.

### Procedure

The research team contacted the principal and teachers of the schools to conduct this study. Parents or legal guardians were informed of the purpose of the study by letter prior to data collection, and written informed consent was required from both participants and their parents/legal guardians. Only students who returned written informed consent signed by parents and themselves participated in this study. The paper-and-pencil questionnaire was administered in a regular classroom by one member of the research team to unify the same protocol. The average time taken to complete the set of questionnaires was approximately 20 min.

### Data analysis

At first step, descriptive statistics were used to examine the average daily time spent in physical activity, recreational screen time, and sleep duration, as well as the adherence to the 24-Hour Movement Guidelines, both separately and for all possible combinations. Sex differences were tested using Student t-test and Chi-squared test for continuous and categorical variables, respectively. A linear regression analysis between 24-Hour Movement Behaviours and interaction sex*predictor was conducted to examine the interaction of sex and each of the movement behaviours (i.e., physical activity, recreational screen time, and sleep duration). As no significant interaction was found between sex and physical activity, recreational screen time, and sleep duration in relation to academic performance (*p* > 0.01), all analyses were performed on the entire sample.

For the main analysis, mixed models were used to examine the association between 24-Hour Movement Behaviours and academic performance. Three separate models for each movement behaviour (i.e., physical activity, recreational screen time, and sleep duration) were estimated. For all models, the estimation of time as within factor (before vs. 1-year after Covid-19 pandemic) and a predictor (i.e., physical activity, recreational screen time, and sleep duration) were included, as well as the interaction time*predictor. A significant effect indicates that the association between movement behaviours and academic performance was statistically different in terms of time (i.e., before vs. 1-year after Covid-19 pandemic). In all models, age, sex, and BMI were included as covariates. All analyses were performed using SPSS version 23.0 for Windows (IBM, Armonk, New York). The level of significance was set at *p* < 0.05.

## Results

Table 1 shows the participant’s characteristics, 24-Hour Movement Behaviours, and academic performance before (T1) and 1-year after (T2) Covid-19 pandemic. Overall, adolescents evaluated before Covid-19 pandemic reported higher physical activity levels, sleep duration, and higher academic performance, as well as lower recreational screen time, compared to adolescents assessed after 1-year into the Covid-19 pandemic (all, *p* < 0.05). In addition, a significantly larger proportion of adolescents, examined before Covid-19 pandemic, met the 24-Hour Movement Guidelines for physical activity, recreational screen time, and sleep duration, independently and all together.

**Table 1:** Descriptive characteristics, prevalence of 24-Hour Movement Behaviours, and academic performance of the two-subsamples of participants before lockdown (T1) and 1-year after (T2) into the Covid-19 pandemic.

Study variables	March to June 2018	February to March 2021	*p*
	M ± SD	M ± SD	
*n* (%)	844 (55.5)	501 (44.5)	
Age groups	13.12 ± 0.86	14.39 ± 1.16	< 0.001
**Sex**			
Boys *n* (%)	484 (57.30)	360 (44.70)	
Girls *n* (%)	224 (42.70)	277 (55.30)	
BMI (kg/m^2^)	21.33 ± 3.86	19.94 ± 3.36	< 0.001
Physical activity (1–5)	2.54 ± 0.61	2.37 ± 0.53	< 0.001
Recreational screen time (h/day)	4.52 ± 2.25	5.45 ± 3.49	< 0.001
Sleep duration (h/day)	8.66 ± 0.96	7.10 ± 0.41	< 0.001
Academic performance (0 – 10)	7.16 ± 1.61	6.89 ± 1.64	< 0.01
Not meeting recommendations *n* (%)	244 (28.90)	332 (66.30)	< 0.001
**Physical activity recommendations *n* (%)**			
Not meeting	547 (64.80)	384 (76.60)	< 0.001
Meeting	297 (35.20)	117 (23.40)	
**Screen time recommendations ***n* (%)			
Not meeting	733 (86.80)	455 (90.80)	< 0.01
Meeting	111 (13.20)	46 (9.20)	
**Sleep duration recommendations** ***n* (%)**			
Not meeting	418 (49.50)	481 (96.00)	< 0.001
Meeting	426 (50.50)	20 (4.00)	
Meeting only one recommendations *n* (%)	394 (46.70)	178 (21.10)	< 0.001
Meeting only two recommendations *n* (%)	156 (31.10)	12 (2.40)	< 0.001
Meeting only three recommendations *n* (%)	28 (3.30)	1 (0.20)	< 0.001

Differences in the study variables between measurements taken in March to June 2018 and February to March 2021 were tested by Student t-test and Chi-squared test for continuous and categorical variables, respectively. *BMI* Body Mass Index.

Table 2 shows the associations between 24-Hour Movement Behaviours and academic performance before and 1-year after Covid-19 pandemic. In each model, the intercept, the covariate (i.e., sex, age, and BMI), the within factor (time: before vs. 1-year after Covid-19 pandemic), the predictor (i.e., physical activity, recreational screen time, or sleep duration) and the interaction time*predictor effects were included. Covariates and predictors were included as z-scores. Intercept represents an estimate of academic performance when all predictors are zero (i.e., an estimate of academic performance when all predictors are zero before Covid-19 pandemic). Time rows represent the difference in academic performance when comparing before and 1-year after into the Covid-19 pandemic. The predictor row shows the degree of association between the predictor (i.e., physical activity or recreational screen time or sleep duration) and academic performance before Covid-19 pandemic. Finally, the interaction rows represent the difference in the slope after closure compared to the slope before closure (i.e., the effect of closure on the association between predictors and academic performance).

**Table 2:** Mixed models analysing the association between 24-Hour Movement Behaviours (physical activity, recreational screen time, and sleep duration) and academic performance before (T1) and 1-year after (T2) into the Covid-19 pandemic.

	Physical activity			Recreational screen time			Sleep duration		
	β	SE	*p*	β	SE	*p*	β	SE	*p*
Intercept	6.94	0.23	< 0.001	7.10	0.12	< 0.001	4.99	0.48	< 0.001
**Covariates**									
Sex	0.18	0.05	< 0.001	0.16	0.06	0.001	0.16	0.05	0.001
Age	− 0.43	0.06	< 0.001	− 0.41	0.05	< 0.001	− 0.40	0.06	< 0.001
BMI	− 0.12	0.05	0.010	− 0.12	0.20	0.009	− 0.12	0.05	0.011
**Within factor**									
Time	− 0.19	0.12	< 0.010	− 0.05	0.20	0.804	1.40	1.72	0.416
**Predictors**									
PA, ST, or SD	0.24	0.12	0.112	− 0.03	0.02	0.239	0.23	0.06	< 0.001
**Interaction**									
Time*physical activity	− 0.40	0.11	< 0.001	–	–	–	–	–	–
Time*screen time	–	–	–	− 0.02	0.03	0.513	–	–	–
Time*slee*p* duration	–	–	–	–	–	–	− 0.18	0.24	0.456

The reported β values are standardized coefficients. *BMI* Body Mass Index; *PA* Physical activity; *ST* Screen time; *SD* Sleep duration. Intercept = Academic performance before Covid-19 lockdown; Time: -1 = March to June 2018 and 1 = February to March 2021; Predictors = Slope before Covid-19 lockdown; Interaction = Slope before Covid-19 lockdown versus slope 1-year after Covid-19 pandemic.

**Figure 2:**
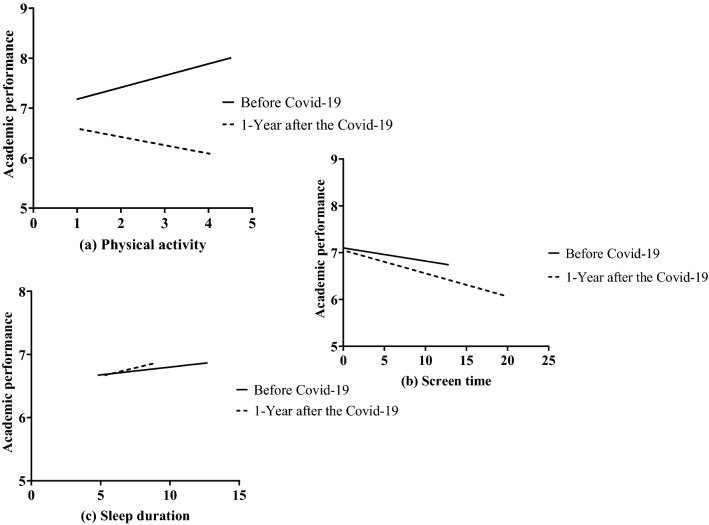
Independent association between physical activity, recreational screen time and sleep duration with academic performance before and 1 year after Covid-19.

Figure 2 shows the independent association of physical activity, recreational screen time, and sleep duration with academic performance before and 1-year after Covid-19. After controlling for the effect of different covariates (age, sex, and BMI), (see Fig. 2, graph a), physical activity was positively and significantly associated with academic performance (β = 0.24; *p* < 0.001) among adolescents assessed before Covid-19 pandemic. For each one-unit increase in physical activity, the estimation of academic performance would increase by 0.24. However, the association became negative (β = 0.24 + ( − 0.40) =  − 0.26; *p* < 0.001) among adolescents evaluated 1-year after Covid-19, that is, physical activity was negatively associated with academic performance, and differences in slopes were statistically significant (*p* < 0.001).

Recreational screen time was not associated with academic performance (β =  − 0.03; *p* > 0.05) among adolescents assessed before Covid-19 pandemic (see Fig. 2, graph b). The interaction effect was not statistically significant, so the association between recreational screen time and academic performance remained non-significant among adolescents evaluated 1-year after Covid-19 (β = − 0.03 + ( − 0.02) =  − 0.05; *p* > 0.05).

Lastly, as displayed in Table 2 and Fig. 2 (graph c), sleep duration was positively and significantly associated with academic performance in the sample of adolescents assessed before Covid-19 pandemic (β = 0.23; *p* < 0.001). The interaction effect was not significant (*p* > 0.05), that is, this association remained significant for adolescents evaluated 1-year after Covid-19 pandemic (β = 0.23 + ( − 0.18) = 0.05; *p* > 0.05).

## Discussion

The first aim of this study was to examine possible changes in 24-Hour Movement Guidelines for adolescents before (T1) and after 1-year (T2) into the Covid-19 pandemic. The second aim examined the possible changes in the relationship between 24-Hour Movement Behaviours and academic performance, before and after 1-year into the Covid-19 pandemic. The main findings of this study are as follows: 1) 24-Hour Movement Behaviours appear to have worsened among young people after 1-year into the Covid-19 pandemic compared to pre-pandemic; 2) adherence to the three 24-Hour Movement Guidelines seem to be significantly lower among Spanish adolescents after 1-year into the Covid-19 pandemic, particularly sleep duration recommendations; 3) the positive relationship between physical activity and academic performance seems to have disappeared 1-year after Covid-19 pandemic; (4) the non-significant relationship between recreational screen time and academic performance and the positive relationship between sleep duration and academic performance does not seem to have changed 1-year after Covid-19.

With respect to the first study hypothesis, it was postulated that adherence to 24-Hour Movement Guidelines would be lower in adolescents assessed after 1-year into the Covid-19 pandemic. Before the Covid-19 pandemic, the adherence to the recommendations was 35.2% for physical activity, 13.2% for recreational screen time, and 50.5% for sleep duration among Spanish adolescents. Only 3.3% met all three 24-Hour Movement Guidelines, while 28.9% did not met with any of the three recommendations. These results are worrying because of the low compliance of Spanish adolescents with the recommendations prior to the Covid-19 pandemic. However, these values seem to have worsened 1-year after Covid-19 pandemic among young people, as significant changes were found in physical activity levels (− 0.17 in the physical activity index), recreational screen time (+ 0.93 h/day) and sleep duration (− 0.56 h/day). Similarly, fewer adolescents seem to meet the recommendations for physical activity (23.4%), recreational screen time (9.2%), and sleep duration (4.0%). Only 0.2% reported meeting all three 24-Hour Movement Guidelines, while 66.3% did not meet any of the three recommendations. The only study published to date that examined changes of 24-Hour Movement Guidelines before and 1-year after into the Covid-19 pandemic is consistent with our results^20^. However, in contrast to Dubuc's study^20^, our study also found significantly lower adherence to screen recommendations among young people. The decrease in physical activity and increase in recreational screen time could partly be explained by Covid-19-related restrictions in Spain during second data collection (e.g., social restrictions, "stay-at-home" recommendations, closure of structured activities, etc.). Although these public health restrictions have been able to reduce the spread of Covid-19, they may also have negatively affected physical activity levels and recreational screen time. Particularly, in this study, sleep recommendations seem to be the most affected 1-year after the Covid-19 pandemic. This could be explained by the increase in recreational screen time, due to the 24-h cycle^39^. It is also possible that the anxiety and depression caused by Covid-19 may have affected sleep quality, which may have a downstream effect on adolescents' sleep duration^40,41^. Therefore, this research suggests that the direct and indirect effects of the Covid-19 pandemic have negatively affected physical activity, recreational screen time, and sleep duration 1-year after into the Covid-19 pandemic.

Regarding the second hypothesis, it was postulated that the relationship between 24-Hour Movement Behaviours and academic performance could have changed 1-year after Covid-19 pandemic. The results showed a positive relationship between physical activity and academic performance before the Covid-19 pandemic, whereas this relationship became negative among adolescents evaluated 1-year after Covid-19. The positive effect of physical activity on the brain may be the result of several factors such as increased cerebral blood flow, oxygen to the brain, synaptic plasticity activity, and neurotransmitter secretion levels, resulting in increased levels of arousal, attention, and effort, which have a positive impact on cognitive task performance immediately after physical activity^42^. However, restrictive measures during Covid-19 have increased stress and anxiety levels^43^, and limited structured physical activities, which has led to a reduction in physical activity levels, especially in adolescents^16^. The implementation of these restrictive measures, such as safe distance, use of face mask, avoidance of sharing materials, etc., has encouraged new forms and places to participate in physical activity, such as individual physical activity at home^16^. In this sense, it is likely that the lack of social interaction and enjoyment of these activities or that they are not performed outdoors could have impacted on the benefits of physical activity on academic performance^42,44^. However, since the relationship between physical activity and academic performance 1-year after Covid-19 has not been studied in depth, further mix-method studies examining this relationship are needed.

Although the amount of recreational screen time appears to have increased after 1-year into the Covid-19 pandemic, there was no change in the relationship between recreational screen time and academic performance before and after 1-year into Covid-19 pandemic, remaining non-significant. These results suggest that recreational screen time does not seem to have affected young people's academic performance before and after 1-year Covid-19. In line with our results, a systematic review with meta-analysis^25^ found that the amount of time spent using screen-based devices was not associated with academic performance. Specifically, the mentioned systematic review with meta-analysis conducted by Adelantado-Renau et al.^25^ only found that television viewing and video game use were the only two screen-based devices negatively associated with academic performance. These authors suggest that the type of screen-based devices assessed (e.g., TV, video games, computer, mobile phone, tablets, etc.), the purpose (e.g., social communication, online networking, gaming, etc.), and the context in which screen media are used (e.g., educational: doing homework, studying, etc. or recreational: playing video games, etc.) may affect the relationship with academic performance^25^. The fact that our study also included computer and mobile phone use within the recreational screen time may explain the lack of association between these two variables.

Finally, a positive relationship between sleep duration and academic performance was also found, both before and after 1-year into Covid-19 pandemic. It is important to note that previous studies have shown that both quantity and quality sleep are positively related to cognitive improvements such as better memory, attention, and executive control^45^. In addition, longer sleep duration has been shown to have a positive effect on adolescents’ ability to retain learned information and make it accessible in the long-term memory^46^. Given that most teachers in Spain use theoretical tests for grading students, it is possible that Spanish adolescents who get enough sleep are better able to retain information more effectively and, consequently, perform better academically^47^. Therefore, although in the present study sleep duration seems to have decreased after 1-year into the Covid-19 pandemic, it does not seem to have been sufficient to cause changes in its relationship with academic performance.

The present study has some limitations that that should be taken into account in future studies. First, this research uses a repeat cross-sectional design in two sub-samples. Although the two sub-samples were similar in terms of age, sex, and socioeconomic status, this design may lead to bias in the results. On the other hand, it does not allow us to examine the directionality of the relationship between 24-Hour Movement Behaviours and academic performance. Future longitudinal studies are needed to reinforce the findings of this study. Secondly, although Covid-19 has affected the entire world population, the restrictions have been different in different countries. Therefore, the results found cannot be extrapolated to the rest of the countries. Thus, more studies that examined changes in 24-Hour Movement Guidelines after 1–2 years into the Covid-19 pandemic are needed. Thirdly, although all questionnaires for measuring 24-Hour Movement Behaviours are valid and reliable, young people may have overestimated or underestimated the time spent on them. Future studies should use device-based measures to assess these three movement behaviours throughout the 24-h period. Fourthly, it was not possible to assess weight and height in the second subsample due to Covid-19 restrictions in the schools evaluated. In this regard, future studies should assess weight and height through electronic scale with a measuring rod. Finally, the use of qualitative methodology could help to explore some of the reasons why adherence to the 24-Hour Movement Guidelines has declined among young people and its relationship with academic performance.

Despite these limitations, this study has some strengths. This is one of the first studies that examined 24-Hour Movement Guidelines, separately and together, before and 1-year after the Covid-19 pandemic. In addition, this is the first study that compared the relationship between 24-Hour Movement Behaviours and academic performance before and after Covid-19 pandemic in adolescents. Finally, sex, age, and BMI were introduced as covariates in the analyses to avoid possible bias.

## Conclusions

The results suggest that physical activity, recreational screen time, and sleep duration appear to have been negatively affected by the Covid-19 pandemic. Particularly, sleep recommendations seem to be the most affected movement behaviour 1-year after the Covid-19 pandemic. Furthermore, 1-year after the Covid-19 pandemic, the relationship between physical activity and academic performance was negative, while recreational screen time and sleep duration with academic performance did not change compared to before Covid-19 results. The reopening of many schools, playgrounds, parks, and organised sports activities in Spain seems to have been insufficient to mitigate the negative consequences of the Covid-19 lockdown on 24-Hour Movement Behaviours. Therefore, there is a serious public health problem that requires immediate and coordinated action by schools, policy makers, health practitioners, and researchers to mitigate the adverse effects of the pandemic on movement behaviours. Specially, it seems very important to design strategies to increase the duration and quality of sleep of young people. Similarly, reducing Covid-related restrictions on physical activity could have a positive impact on academic performance.

## Data Availability

The datasets generated during and/or analyzed during the current study are available from the corresponding author on reasonable request.
